# Identification and Description of Balance, Mobility, and Gait Assessments Conducted via Telerehabilitation for Individuals With Neurological Conditions: Protocol for a Scoping Review

**DOI:** 10.2196/27186

**Published:** 2021-12-09

**Authors:** Jennifer O'Neil, Keely Barnes, Erin Morgan Donnelly, Lisa Sheehy, Heidi Sveistrup

**Affiliations:** 1 School of Rehabilitation Sciences University of Ottawa Ottawa, ON Canada; 2 Bruyère Research Institute Ottawa, ON Canada; 3 Bayshore Home Health Ottawa, ON Canada

**Keywords:** telerehabilitation, remote assessment, outcome measures, neurology, rehabilitation

## Abstract

**Background:**

The COVID-19 global pandemic pushed many rehabilitation practitioners to pivot their in-person practice to adopt telerehabilitation as their main method of delivery. In addition to documenting information on interventions used with clients, it is best practice for therapists to use reliable and validated outcome measures to inform their interventions.

**Objective:**

Through this scoping review, we aim to identify (1) which outcomes are being used remotely to assess balance, mobility, and gait in patients with neurological conditions, and (2) what psychometric data (validity, reliability, etc.) for remotely administered outcomes are available.

**Methods:**

Three main concepts will be included in our search: (1) neurological conditions; (2) administration by telerehabilitation; and (3) outcome measures for balance, mobility, and gait. Studies reporting remote assessment of neurological conditions published since 1990 will be included. The database search will be completed in MEDLINE (Ovid), CINAHL, PubMed, PsycINFO, EMBASE, and Cochrane. Gray literature including dissertations, conference papers, and protocol papers will also be sourced. Two reviewers will independently screen each title and abstract using pre-established inclusion and exclusion criteria. Manuscripts that appear to meet the criteria will be subject to further review, and full-text extraction using a pre-piloted extraction sheet if all criteria are met. The data will be categorized by assessment types describing impairments (such as balance, strength, and mobility) or activity limitations or participation restriction (such as functional mobility, ambulatory functions, and activities of daily living).

**Results:**

This scoping review will document outcome measures currently used in the remote assessment of neurological conditions. To date, 235 titles and abstracts were screened. We are in the process of finalizing the full text screening for the inclusion of articles. We expect the full screening to be completed in November 2021 and data analysis in January 2022. Our results are expected to be published in early 2022.

**Conclusions:**

The optimal use of telerehabilitation as a mode to deliver rehabilitation intervention should be coupled with the completion of validated outcome measures. Therefore, it is crucial to further our knowledge on remote outcome measures and therapeutic assessments.

**International Registered Report Identifier (IRRID):**

PRR1-10.2196/27186

## Introduction

Telerehabilitation, teletherapy, and virtual rehabilitation are terms describing the use of information and communication technologies including phone or videoconferencing to provide rehabilitation services to people remotely, in their home or other environment [1]. In 2017, Peretti et al [2] reported that the field of telerehabilitation was considerably new but the use had rapidly grown in high-income countries. The COVID-19 global pandemic has only further driven the adoption of this rehabilitation delivery method. Data from the Ontario Telemedicine Network (OTN) [3] documented a 36% growth in virtual visits in their annual 2018-2019 report, totaling over 1 million telemedicine visits. Originally used to reduce travel time and costs for patients and health care providers, as well as to improve access to specialists for rural communities [4], the COVID-19 global pandemic has pushed health care practitioners to adopt telemedicine as their main method of delivery. Similar rapid uptake of telerehabilitation enabled rehabilitation clinicians to continue addressing patient health needs while following public health guidelines.

Remote rehabilitation interventions for a variety of neurological conditions highlight the benefits of telerehabilitation and the need for further research [5-7]. Reviews have systematically demonstrated a positive impact on outcomes including gait, mobility, strength, and daily function in people living with deficits after stroke [8,9], traumatic brain injury [10,11], Parkinson disease [12], and multiple sclerosis [13,14]. By contrast, a scoping review by O’Neil et al [15] reported that there were limited guidelines on the implementation parameters of interventions delivered remotely.

An outcome measure is defined as a clinical tool to objectively measure changes in function of a patient as a result of an intervention [16]. It is best practice for therapists to complete assessments using reliable and validated outcome measures to inform their interventions. Choosing valid and reliable outcome measures is critical in assessing intervention efficacy and meaningful clinical change [16]. While clinicians use outcome measures to guide their interventions, insurance companies require clinicians to objectively document the progress of patients using validated outcome measures to provide credible and reliable justification for treatment. Consequently, not using objective, reliable, valid, and responsive outcome measures could have financial impacts on patients and health care providers alike. More importantly, without use of appropriate outcome measures, clinicians cannot effectively measure the impact of their proposed intervention on targeted impairments, therefore not identifying whether interventions are working for each patient.

Potential barriers such as limited space in the patient’s home, equipment availability, or safety issues may compromise the validity or reliability of the remote outcome measures used by the therapist. Depending on the method used for telerehabilitation, additional restrictions could also impact the choice of outcome measure. For example, when assessing balance, poor visibility via videoconference or the use of phone calls could lead to choosing measures that are less objective, such as patient-reported questionnaires instead of specific clinical outcome measures targeting balance. Ultimately, the use of outcome measures that have not been appropriately tested for reliability and validity will not be able to guide intervention planning and may adversely affect the patient’s recovery.

The use of valid outcome measures regardless of whether a clinician is using in-person or tele-platforms methods is necessary. Previously, a hybrid model of service delivery with outcomes assessments performed before, after, or during telerehabilitation interventions was typically completed in-person; however, due to COVID-19, in-person visits are now curtailed or cancelled. Although there are a wide range of reliable and valid in-person rehabilitation assessments [17], there is a need to systematically review outcome measures performed via telerehabilitation, to recommend the most valid, reliable, acceptable, and safe measures to be administered remotely.

Validity, the ability for a tool to assess what it is intended to assess, and reliability, the ability for the test to be reproduced with similar results, are key features of evidence-based assessments [18]. Mani et al [19] studied assessment techniques using telerehabilitation in a population with musculoskeletal deficits including back pain, ankle and elbow joint disorders, and total knee replacement. Authors from this study concluded that there was good validity and reliability for a variety of remote outcome measures including function (eg, Oswestry Disability Index), range of motion (eg, goniometry), strength (eg, self-resistance), and balance (eg, Tinetti Balance and Gait Assessment). While there are a limited number of validated remote outcome measures for use with the musculoskeletal population, a gap remains regarding telerehabilitation assessments for individuals with neurological conditions. Remote assessments of people living with neurological conditions are limited but have been studied in both the pediatric and adult population. For example, the feasibility and concurrent validity of using the Movement Assessment Battery for Children has been established by Nicola et al in 2018 [20]. The use of smartphone apps, accelerometers, and activity tracking devices to assess activity level and gait parameters in people living with impairments after stroke, brain injury, and multiple sclerosis also has been reported as remote assessment methods in recent reviews [21,22].

This study aims to review the literature to identify and describe (1) outcome measures that are being used remotely to assess balance, mobility, and gait in patients with neurological conditions, and report (2) the available psychometric data (eg, validity, reliability, consistency, equivalence) for these outcome measures when used remotely.

## Methods

### Systematic Search

This scoping review will follow the methodological steps outlined by Arksey and O’Malley [23], and expanded by Colquhoun et al [24]. First, a search strategy will be identified using specific inclusion criteria around the following main concepts: (1) neurological conditions (eg, acquired brain injury, neurodegenerative disorders); (2) administration by telerehabilitation; and (3) clinical outcome measures (eg, postural balance, functional mobility, activity of daily living, gait assessments, motor assessments). Supported by an institutional research librarian, an initial systematic database search was conducted between December 13, 2020, and January 5, 2021, in MEDLINE (Ovid), CINAHL, PubMed, PsycINFO, EMBASE, and Cochrane. The search strategy included MeSH terms and Boolean strategies to clarify the search and identify studies published from 1990 to January 2021. Gray literature including dissertations, conference papers, and references from protocol papers will also be searched using Google Scholar and reference lists of included papers will be hand searched (Multimedia Appendix 1).

### Study Screening and Selection

Two reviewers (KB and EMD) will independently screen each title and abstract using pre-established inclusion and exclusion criteria (Textbox 1). Using the Covidence software (Covidence AS), abstracts meeting all inclusion and exclusion criteria will be selected for full manuscript review. Full texts from selected articles will be screened independently by the same 2 reviewers (KB and EMD) to confirm eligibility before proceeding to data extraction. This screening step will be piloted with 5 selected studies and reviewed by a third reviewer to increase interrater reliability prior to screening all articles. Following each screening step, conflicts will be resolved by a third rater (JO or LS) when necessary. Interrater reliability between the independent reviewers for the full-text review will be documented by reporting the Cohen κ agreement for the included studies. The reference list from each included scoping or systematic review will be manually searched for additional articles.

**Inclusion and exclusion criteria.**

**Inclusion criteria**

*Population with neurological conditions*

- Acquired brain injury (ie, traumatic brain injury, stroke, brain tumors)
- Neurodegenerative disorders (ie, multiple sclerosis, Parkinson disease, amyotrophic lateral sclerosis, motor neuron disease)

*Assessment*

- Tele-platform
- Telerehabilitation (eg, telerehabilitation, virtual rehabilitation, remote rehabilitation)
- Telemedicine (eg, telehealth, eHealth, mHealth, app, text messaging, sensor based)
- Virtual reality (eg, augmented reality, computer simulation)
- Remote consultation (eg, teleconsultation, consultation, remote)
- Telemonitoring (eg, remote monitoring, remote assessment)

*Health professional*

- Allied health occupations, allied health personnel, and rehabilitation therapist (ie, physiotherapist, physical therapist, occupational therapist, speech language pathologist, communication therapist, kinesiologist, athletic therapist, nurse, rehabilitation therapist, psychologist, neuropsychologist, social worker)
- Physiatrist, physicians, physical medicine

*Outcome measures*

- Postural balance assessment (eg, balance, postural, postural control)
- Functional mobility limitation/assessment (eg, functional mobility, transfers, wheelchair mobility). See “Exclusion Criteria”
- Daily function (eg, upper extremity function, fine motor skills, dressing and toileting, communication)
- Gait assessment (eg, neurologic ambulation disorders, ambulation disorders, level of independence, ambulation, gait speed, gait analysis)
- Motor function assessments (eg, strength, range of motion, stage of motor recovery)

*Limits*

- Language: English or French
- Period: 1990-January 2021

**Exclusion criteria**

*Assessment*

- Only in-person assessment

*Outcome measures*

- All other outcome measures not listed in the “Inclusion Criteria.”

*Study design*

- Meta-analysis and reviews (but original papers in the reference list will be searched for inclusion)

### Data Extraction

Data will be extracted using a pre-established data extraction sheet (Multimedia Appendix 2). To ensure the charting process is comprehensive, cohesive, and complete, the extraction sheet will be piloted with a set of articles prior to starting data extraction.

### Quality Appraisal of Included Studies

When possible, a quality appraisal will be completed using the appropriate quality reporting tool proposed by the Equator Network [25]. As such, randomized controlled trials will be assessed with CONSORT (Consolidated Standards Of Reporting Trials), case reports with CARE (CAse REports), observational studies with STROBE (STrengthening the Reporting of OBservational studies in Epidemiology), and qualitative studies with the SRQR (Standards for Reporting Qualitative Research). Overall, the PRISMA-ScR (PRISMA Extension for Scoping Reviews) checklist will be used to ensure proper reporting of this proposed scoping review [26].

### Data Analysis and Interpretation

Once data extraction is complete, the information will be collated and synthesized following a systematic approach. Guided by the International Classification of Function (ICF), data will be categorized using various ICF domains. For example, outcome measures describing impairments of body structures will include measures such as balance and strength, while outcome measures assessing activities and participation will include functional mobility, transfers, ambulation, and activities of daily living [27]. This list of remote outcome measures will also include the available psychometric data including information around validity (eg, content, construct, convergent), reliability (eg, stability [intraclass correlation coefficient], consistency [Cronbach α], equivalence [α]).

Once synthesized, data will be subcategorized by types of regulated health care professionals to facilitate clinical usefulness. The subcategories will further be classified by the type of telehealth platform used. Finally, field consultation will be completed by asking 5 allied health professionals to review the interpretation table to validate the clinical usefulness and potential gaps in the interpretation of our findings.

## Results

To date, the initial systematic search has been completed and 293 studies were imported for screening. After removing 58 duplicates, 235 titles and abstracts were screened. We are in the process of finalizing the full text screening for the inclusion of articles. We expect the screening to be completed in November 2021 and data analysis in January 2022. Our results are expected to be published in early 2022.

## Discussion

Telerehabilitation can not only improve access for individuals who may not benefit from traditional in-person services, but it may also have financial benefits, reducing costs for the health care system, health care provider, and patient [2]. The optimal use of telerehabilitation as a mode to deliver rehabilitation interventions should be coupled with the completion of valid outcome measures. Therefore, it is crucial to further our knowledge on remote outcome measures and therapeutic assessments. Our findings could influence clinical practice and patient care and guide clinical research in telerehabilitation. This scoping review will help determine how remote assessments are currently being conducted and provide information on the valid and reliable measures currently available. Furthermore, results from this study will allow recommendations to be made for what assessments or areas of remote assessment need to be further validated.

## Appendix

Multimedia Appendix 1 Search strategy.

Multimedia Appendix 2 Data extraction.

## Abbreviations

CARE: CAse Reports
CONSORT: Consolidated Standards Of Reporting Trials
ICF: International Classification of Function
OTN: Ontario Telemedicine Network
PRISMA-ScR: PRISMA Extension for Scoping Reviews
SRQR: Standards for Reporting Qualitative Research
STROBE: STrengthening the Reporting of OBservational studies in Epidemiology
