# Supplementary material for: Identification and Description of Balance, Mobility, and Gait Assessments Conducted via Telerehabilitation for Individuals With Neurological Conditions: Protocol for a Scoping Review
Source: JMIR Res Protoc. 2021 Dec 9;10(12):e27186. doi: 10.2196/27186 (PMC8704120; doi:10.2196/27186)
Supplement: Multimedia Appendix 1 [file resprot_v10i12e27186_app1.docx]

**Appendix A**

**Search Strategy**

| **Major concepts** | | |
| --- | --- | --- |
| Identify the main concepts of your research topic. | | |
| 1. Telehealth | 2. Neurologic Conditions | 3. Rehabilitation Assessment/Evaluation |

| **Search Strategy** | | | | |
| --- | --- | --- | --- | --- |
|  | | | | |
|  | **Concept 1 AND Concept 2 AND Concept 3** | | | |
|  | Telehealth | Neurologic | Rehabilitation Outcome measures/ Assessments/ Evaluation |  |
| **OR** | Tele-rehabilitation (MeSh: Rehabilitation, Vocational) | Neurologic ambulation disorders | Gait/ambulation/walk Outcome measures/ Assessments/ Evaluation |  |
| **OR** | Virtual rehabilitation | Ambulation/Gait disorders | Motor function Outcome measures/ Assessments/ Evaluation |  |
| **OR** | Remote rehabilitation | Acquired brain injury or ABI | Balance Outcome measures/ Assessments/ Evaluation |  |
| **OR** | Telemedicine (MeSh: Telepathology, Teleradiology, Telerehabilitation) | Traumatic brain injury or Brain injury or TBI | Mobility Outcome measures/ Assessments/ Evaluation |  |
| **OR** | Mobile health | Stroke | Sitting or standing Posture Outcome measures/ Assessments/ Evaluation |  |
| **OR** | Telerehabilitation | Brain Tumors | Daily Function Outcome measures/ Assessments/ Evaluation |  |
| **OR** | eHealth | Neurodegenerative | Strength assessment/evaluation |  |
| **OR** | mHealth | Parkinson |  |  |
| **OR** | Remote consultation | Multiple Sclerosis |  |  |
| **OR** | Telemetry (MeSh: Remote sensing technology) | Cerebral palsy |  |  |
| **OR** | Telecommunication (MeSh: Electronic mail, Radar, Radio | Spinal cord injury, SCI |  |  |
| **OR** | Telemonitoring |  |  |  |
| **OR** | Telecare |  |  |  |
| **OR** | Videoconferencing (MeSh: Webcasts as topic, Wireless technology) |  |  |  |
| **OR** | Videoconsultation |  |  |  |
| **OR** | Distant care |  |  |  |
| **OR** | Distant rehabilitation |  |  |  |
| **OR** | Distant therapy |  |  |  |
| **OR** | Distant physio |  |  |  |
| **OR** | Distant consultation |  |  |  |
| **OR** | Virtual reality (MeSh: Augmented reality, molecular docking simulation, molecular dynamic simulation, patient-specific modeling, computer simulation) |  |  |  |
| **OR** | Teleconcussion |  |  |  |
| **OR** | Telestroke |  |  |  |
| **OR** | Telepractice |  |  |  |
| **OR** | Tele-speech |  |  |  |
| **OR** | Teleconsultation |  |  |  |
| **OR** | Tele-OT |  |  |  |
