# Supplementary material for: Identification and Description of Balance, Mobility, and Gait Assessments Conducted via Telerehabilitation for Individuals With Neurological Conditions: Protocol for a Scoping Review
Source: JMIR Res Protoc. 2021 Dec 9;10(12):e27186. doi: 10.2196/27186 (PMC8704120; doi:10.2196/27186)
Supplement: Multimedia Appendix 2 [file resprot_v10i12e27186_app2.docx]

**Appendix B Data Extraction Sheet**

# Step 1: Study Selection- Title and Abstract Review

| Review title or ID |  |
| --- | --- |
| Study ID (surname of first author and year first full report of study was published (e.g., Smith 2001)  Add contact details of corresponding authors |  |

# General Information

| Date form completed (dd/mm/yyyy) |  |
| --- | --- |
| Name/ID of person extracting data |  |
| Reference citation |  |
| Publication type  (e.g., full report, abstract, letter) |  |
| Notes: | |
| Abstract | |

# Study Eligibility

| Study Characteristics | Eligibility criteria  (Insert inclusion criteria for each characteristic as defined in the Protocol- highlight when necessary) | | Eligibility criteria met ? | | | Notes |
| --- | --- | --- | --- | --- | --- | --- |
|  |  |  | Yes | No | Unclear |  |
| Type of intervention | Telerehabilitation/remote assessment/tele-assessment/rehabilitation assessment/other  In-person | |  |  |  |  |
| Neurological Population | A) ABI (CVA/Stroke, TBI, Brain Tumor, CP)  B) Neurodegenerative (PD, MS)  c) Spinal cord Injury (SCI) | |  |  |  | Insert category here  (A, B, C) |
| Type of study | RCT, Case study, Case-control study, Reviews, Meta-analysis, Cohort Study, Feasibility Study Other______________ | |  |  |  |  |
| Age group | 18-64  65+  17 under | |  |  |  |  |
| Location | Country: | |  |  |  |  |
| Year of publication | 1990-2021 | |  |  |  |  |
| Language | English or French  Others: | |  |  |  |  |
| Main objectives   - Objective: - Assessment studied: | | | | | | |
| Abstract and title screening  INCLUDE  EXCLUDE  Full Text screening  INCLUDE  EXCLUDE | |  | | | | |
| Reason for exclusion |  | | | | | |
| Notes: | | | | | | |

DO NOT PROCEED TO FULL TEXT REVIEW IF STUDY EXCLUDED FROM REVIEW

# Step 2: Full Text Review – Data Extraction

| **Study information not captured in first selection round (title and abstract)** | |
| --- | --- |
| Sample size |  |
| Study design |  |
| Main Findings   - Validated - Reliable - Feasibility - Safety |  |

| **AIM 1: Which outcome measures are being used remotely to assess balance, mobility and gait in patients with neurological conditions.** | |
| --- | --- |
| Name of Outcome Measures and Type of Outcome (observational, timed, distance, self-report) |  |
| ICF domains  1- body structures: balance, strength or  2- activities and participation: functional mobility, transfers, and ambulatory functions) |  |
| Assessment teleplatform (synchronous/asynchronous, phone, videoconference, VR, APP, text messaging, sensor-based) |  |
| Neurological conditions |  |
| Rehabilitation professional |  |
| **AIM 2: Reported outcome measure psychometric data (validity, reliability) if available** | |
| a) Name of Outcome Measures  b) Available psychometric data  Validity – content, criterion (predictive/concurrent), construct  Reliability – test/retest, inter-rater, intra-rater  Standard Error of Measurement  Minimal Detectable Change  Minimal Clinically Important Difference  Cut-off Scores  Normative Data  Responsiveness (Effect size, Standardized Response Mean)  Internal Consistency  Floor/Ceiling Effects |  |
| Additional information (i.e., safety, resources, cost...) |  |
| Notes and Additional Information: | |
